# Predictors of Acute Chest Syndrome Following Vaso-Occlusive Crisis in Pediatric Sickle Cell Disease

**DOI:** 10.3390/diagnostics16121875

**Published:** 2026-06-16

**Authors:** Narcisse Elenga, Noelis Thomas Boizan, Emmanuel Irakoze, Mody Diop, Gabriel Bafunyembaka

**Affiliations:** Sickle Cell Reference Center, University Hospital of French Guiana, 3 Avenue Alexis Blaise, Cayenne 97300, French Guiana; noelis.thomas@ch-cayenne.fr (N.T.B.); emmanuel.irakoze@ch-cayenne.fr (E.I.); mody.diop@ch-cayenne.fr (M.D.); gbafunyembaka@gmail.com (G.B.)

**Keywords:** sickle cell disease, acute chest syndrome, vaso-occlusive crisis, pediatrics, risk factors, predictive model, French Guiana

## Abstract

**Background/Objectives**: Acute chest syndrome (ACS) is a frequent and potentially life-threatening complication of sickle cell disease (SCD) that often develops during hospitalization for vaso-occlusive crisis (VOC). The early identification of pediatric patients at risk remains challenging, particularly in high-prevalence settings. This study aimed to identify predictors of acute chest syndrome following vaso-occlusive crisis in children with SCD. **Methods**: We conducted a retrospective cohort study of children and adolescents (≤18 years) with confirmed SCD admitted for VOC to Cayenne Hospital Center, French Guiana, between January 2014 and September 2024. ACS that occurred during hospitalization or within 7 days of admission was recorded. Multivariable logistic regression was used to identify independent predictors, and model performance was assessed using receiver operating characteristic (ROC) analysis. **Results:** Among the 825 VOC episodes in 190 patients, 239 (29%) were complicated by ACS. Independent ACS predictors were thoracic or abdominal pain at presentation (adjusted odds ratio [aOR] 14, 95% CI 6–32, *p* < 0.001), prior history of ACS (aOR 7.4, 95% CI 4.5–12.1), and Hb SS or Sβ^0^ genotype (aOR 1.8, 95% CI 1.2–2.4), age > 10 years (aOR 1.6, 95% CI 1.1–2.4), male sex (aOR 1.6, 95% CI 1.1–2.4), Hydroxyurea treatment was associated with a higher risk of acute chest syndrome (aOR 8.7, 95% CI 5.2–14.5), likely reflecting greater baseline disease severity among treated patients. The probability threshold maximizing the Youden index was 0.67, corresponding to a Youden index of 0.56. At this threshold, the model had a sensitivity of 61%, a specificity of 95%, a positive predictive value of 85%, and a negative predictive value of 86%. The apparent area under the receiver operating characteristic curve was 0.87 (95% CI, 0.83–0.89). The receiver operating characteristic analysis yielded an area under the curve of 0.87, indicating good apparent discrimination. **Conclusions:** These findings support targeted monitoring and early preventive strategies during pediatric VOC admissions.

## 1. Introduction

Sickle cell disease (SCD) is the most common monogenic genetic disorder worldwide, affecting more than 5 million individuals, with approximately 300,000 births affected each year globally [1]. In France, SCD is the most frequent rare disease, with an estimated incidence of 1 in 1900 live births, corresponding to 300–350 newborns diagnosed with major sickle cell syndromes annually [2].

SCD is an autosomal recessive hemoglobinopathy caused by a single-point mutation in the β-globin gene on chromosome 11; glutamic acid is replaced by valine at position six (Glu6Val), resulting in the production of abnormal hemoglobin S (HbS). Under conditions such as hypoxia, dehydration, acidosis, or hyperthermia, HbS polymerizes once a critical concentration of deoxygenated HbS is reached, leading to red blood cell sickling. Although this process is initially reversible upon reoxygenation, repeated cycles result in irreversible erythrocyte damage, reduced red blood cell deformability, chronic hemolysis, and anemia [3].

Clinically significant disease occurs in individuals who are homozygous for HbS (Hb SS) or compound heterozygous for HbS and another abnormal hemoglobin, including Hb SC and Hb S/β-thalassemia. Individuals carrying a single abnormal allele (sickle cell trait) are generally asymptomatic but remain potential carriers. Primarily affecting the postcapillary microcirculation, vaso-occlusion results from reduced erythrocyte deformability, increased adhesion to the vascular endothelium, and a proinflammatory and prothrombotic milieu. As a systemic disorder, SCD can affect virtually all vascularized organs [3].

Acute SCD complications include vaso-occlusive crisis (VOC), also referred to as vaso-occlusive episode (VOE), acute chest syndrome (ACS), stroke, and priapism, all of which constitute medical emergencies. Among these, ACS is one of the most severe complications and remains a leading cause of morbidity and mortality in children with SCD. More than 50% of children with homozygous sickle cell disease (Hb SS) experience at least one episode of acute chest syndrome during the first decade of life [3]. Although it predominantly affects patients with the Hb SS genotype, ACS also occurs in individuals with Hb SC disease and HbS/β-thalassemia. The pathophysiology of ACS is multifactorial and may involve pulmonary microvascular occlusion due to in situ sickling; infectious processes, particularly in children; atelectasis secondary to rib or vertebral infarction; and fat embolism resulting from bone marrow necrosis [4].

ACS may present as an inaugural manifestation of SCD or, more commonly, as a complication of a VOC, particularly thoracic or abdominal VOC. By definition, ACS is characterized by the appearance of a new pulmonary infiltrate on chest imaging associated with one or more clinical features, including fever, respiratory symptoms, hypoxemia, or thoracoabdominal pain [5].

Several factors have been associated with an increased ACS risk, including severe pain episodes, abdominal or thoracic VOC, recent surgery, infections, and iatrogenic factors such as corticosteroid exposure or excessive opioid administration [6]. However, predictors of progression from VOC to ACS remain incompletely defined, particularly in pediatric populations and in specific geographic and epidemiological contexts [7].

In French Guiana, SCD represents a major public health concern. The incidence of major sickle cell syndromes at birth is estimated at 1 in 227, with more than 10% of the population carrying the sickle cell trait [8], compared with approximately 2.7% in mainland France [9]. This high prevalence is associated with a substantial burden in terms of hospitalizations, notably for acute complications such as VOC and ACS [10].

Identifying predictive factors for ACS development following VOC in children with SCD is therefore crucial for improving early risk stratification, optimizing clinical management, and potentially reducing morbidity and length of hospital stay.

The primary objective of this study was to identify clinical and biological predictors of progression from VOC to ACS among pediatric patients with SCD hospitalized at the Cayenne Hospital Center. Secondary objectives were to describe the demographic and clinical characteristics of pediatric patients with ACS in French Guiana—including age, sex, genotype, glucose-6-phosphate dehydrogenase deficiency, history of asthma, prior surgical procedures, hydroxyurea therapy, and recent VOC history—and to compare the length of hospital stay between admissions for uncomplicated VOC and those complicated by subsequent ACS.

## 2. Methods

### 2.1. Study Design and Setting

We conducted a retrospective cohort study at the Cayenne Hospital Center, the main tertiary care facility for SCD in French Guiana. The study period spanned from 1 January 2014 to 15 September 2024.

### 2.2. Study Population

All children and adolescents aged 6 months to 18 years with a confirmed diagnosis of SCD who presented to the emergency department (ED visits) during the study period were eligible for inclusion.

### 2.3. SCD Diagnosis and Genotyping

Universal neonatal SCD screening was implemented, with diagnosis confirmed at 6 months of age using hemoglobin electrophoresis or high-performance liquid chromatography, complemented by sickle cell genotyping.

### 2.4. Inclusion Criteria

- Age ≤ 18 years at hospitalization;
- Confirmed SCD diagnosis;
- Admission to the ED for VOC.

### 2.5. Exclusion Criteria

- Presence of ACS at the time of admission;
- Incomplete medical records preventing outcome assessment;
- Chronic lung disease unrelated to SCD.

Each VOC-related admission was considered an independent observation. When multiple such admissions occurred in the same patient, all eligible episodes were included in the main analysis.

### 2.6. Definition [11]

- Vaso-occlusive crisis: An acute, painful episode requiring hospital admission and not attributable to another identifiable cause.
- Acute chest syndrome: The occurrence of a new pulmonary infiltrate on chest imaging associated with at least one clinical feature—fever, respiratory symptoms, hypoxemia, or thoracoabdominal pain—either during hospitalization or within 7 days following VOC admission.

### 2.7. Data Collection

Data were retrospectively extracted from electronic and paper medical records using a standardized data collection form. Variables included demographics, SCD-related characteristics, comorbidities, chronic treatments, VOC characteristics, laboratory parameters at admission, hospital course, and outcomes.

Because multiple VOC episodes could occur in the same patient, a sensitivity analysis restricted to the first episode per individual was performed to account for within-subject correlation. All eligible VOC episodes were included in the primary episode-level analysis. Because some patients contributed more than one episode, the independence assumption of conventional logistic regression may not have been fully satisfied. A sensitivity analysis restricted to one eligible episode per patient was therefore performed. A valid clustered analysis using generalized estimating equations could not be conducted because a unique patient identifier linking all episodes was not available in the final analytical dataset.

### 2.8. Sample Size Calculation

The minimum sample size was estimated assuming an expected ACS incidence of 20% [11] among VOC episodes, a two-sided alpha of 0.05, and 80% power. Approximately 246 VOC episodes were required, ensuring at least 10 outcome events per predictor for multivariable logistic regression.

### 2.9. Statistical Analysis

Continuous variables are summarized as means ± standard deviations or medians with interquartile ranges, as appropriate. Categorical variables are expressed as frequencies and percentages. Comparisons were performed using Student’s *t*-test or Mann–Whitney U test for continuous variables and χ^2^ or Fisher’s exact test for categorical variables.

Univariate logistic regression was used to evaluate potential predictors of progression from VOC to ACS. Variables with *p* < 0.20 and those deemed clinically relevant were included in a multivariable logistic regression model. Adjusted odds ratios (aORs) with 95% confidence intervals (Cis) were reported. Model discrimination was assessed using receiver operating characteristic (ROC) curve analysis, and the area under the ROC curve was reported with its 95% confidence interval. The optimal probability threshold was determined by maximizing the Youden index, defined as sensitivity + specificity – 1. At this threshold, sensitivity, specificity, positive predictive value, and negative predictive value were calculated. Statistical significance was set at *p* < 0.05 and analyses were performed using STATA version 16.0.

### 2.10. Ethical Considerations

This study was conducted in accordance with French regulations governing research that does not involve human subjects [12] and complied with the Reference Methodology MR-004. The Cayenne Hospital Center (Centre Hospitalier de Cayenne) declared compliance with MR-004 on 25 September 2023. A data protection impact assessment was performed, and a summary of the study was made publicly available on the Health Data Hub website No 20238540.

The legal basis for data processing was carrying out a task in the public interest. All data were derived from patients’ medical records and collected as part of routine clinical care. Prior to analysis, the study database was pseudonymized.

Patients or their legal representatives were informed of the study objectives and of their right to object to the use of their data or to withdraw from the study at any time prior to database locking, without consequence. Individual information notices were sent by post. In the absence of an expressed objection within one month of mailing, participation was considered not opposed, in accordance with MR-004 requirements.

Given the high rate of loss to follow-up in French Guiana, partly due to population mobility, participants were included by default when no response was received within the specified timeframe or when mailed notices were returned as undeliverable. Deceased patients who had not objected to the use of their data during their lifetime were also included.

Participants or their legal representatives could exercise their right to object using the enclosed objection form or by contacting the study sponsor or the institutional Data Protection Officer.

General information about the study was provided through notices posted in the relevant hospital departments and through the hospital’s patient information materials.

## 3. Results

### 3.1. Study Population

Between 1 January 2014 and 15 September 2024, a total of 913 VOC hospitalizations in 203 pediatric patients with SCD were identified, out of a total of 165,000 pediatric ED visits. After applying the exclusion criteria (ACS at admission, incomplete records, or unrelated chronic lung disease), 825 VOC episodes in 190 patients were included in the final analysis.

Regarding patient demographics (Table 1), 60% of patients were older than 10 years, with a median age of 12 years (range, 8–16). Females accounted for 51.5% of the cohort, and 68% of patients experienced multiple hospital admissions during the study period; in particular, 40% of patients had an average of one to two hospital admissions per year due to VOC. The frequency of hospital admissions was not significantly associated with ACS incidence following admission. Hemoglobin SS was the most prevalent genotype (74%), and 53% [101/190] of patients were receiving hydroxyurea prior to admission. Regarding past medical history, 17% had asthma, 2.3% had congenital heart disease, 3.2% had epilepsy, 28.4% had a previous history of ACS, and 15% had a recent upper respiratory tract infection (URTI). Glucose-6-phosphate dehydrogenase deficiency was identified in 10% of patients.

### 3.2. ACS Incidence

Out of 825 VOC episodes, 239 (29%) were complicated by ACS either during hospitalization or within 7 days after admission (Table 1). The median time from VOC admission to ACS onset was 3 days (interquartile range [IQR], 2–5).

### 3.3. Predictive Factors for ACS (Table 1)

Patients who developed ACS were more likely to present with thoracic or abdominal pain during VOC (97% vs. 60%, *p* < 0.001), have a prior history of ACS (51% vs. 19%, *p* < 0.001), be older than 10 years (80% vs. 53%, *p* < 0.001), and have the Hb SS/S/ß° genotype (76% vs. 68%, *p* < 0.001). In addition, HU treatment prior to admission was associated with a higher risk of acute chest syndrome (aOR 8.7, 95% CI 5.2–14.5). Low baseline hemoglobin also showed a trend toward an increased risk, but did not reach statistical significance. Table 2 and Table 3 present the detailed multivariate analyses of the predictive factors for ACS following a VOC.

### 3.4. Sensitivity Analysis

When including only the first VOC episode per patient (n = 190), the results were consistent. The adjusted odds ratios reported in this section were derived from the sensitivity analysis and therefore differ slightly from those presented in Table 3, which correspond to the primary multivariable model.

- Thoracic/abdominal pain VOC: aOR 2.88 (95% CI: 1.45–5.72).
- History of ACS: aOR 2.2 (95% CI: 1.28–2.9).
- Hb SS/S/ß° genotype: aOR 1.91 (95% CI: 1.3–2.6).
- Hydroxyurea therapy: aOR 5.2 (95% CI: 4.3–9.6).
- Male sex: aOR 1.6 (95% CI: 1.2–2.4).
- Age > 0 years: aOR 1.6 (95% CI: 1.1–2.4).

This confirms the robustness of the identified predictive factors.

**Figure 1:**
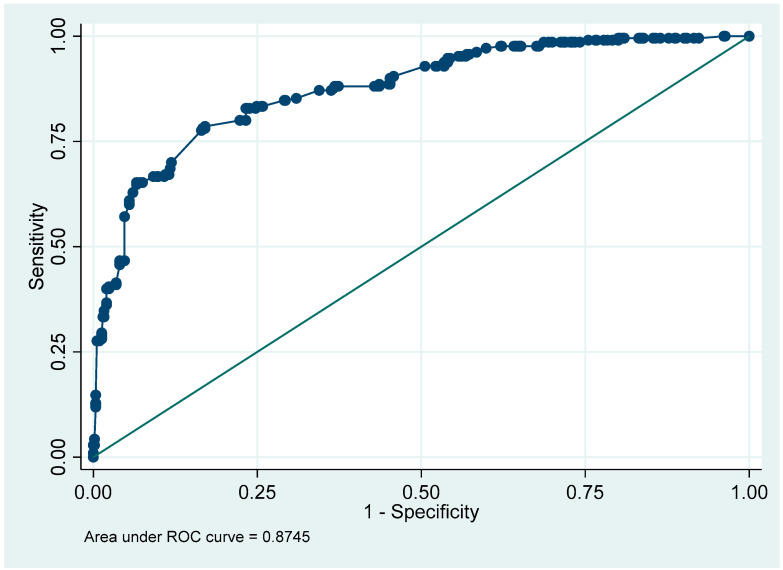
ROC curve of our model for predicting ACS following a VOC.

The probability threshold maximizing the Youden index was 0.67, corresponding to a Youden index of 0.56. At this threshold, the model had a sensitivity of 61%, a specificity of 95%, a positive predictive value of 85%, and a negative predictive value of 86%. The apparent area under the receiver operating characteristic curve was 0.87 (95% CI, 0.83–0.89) (Figure 1). The receiver operating characteristic analysis yielded an area under the curve of 0.87, indicating good apparent discrimination.

## 4. Discussion

In this retrospective cohort study of pediatric patients with SCD admitted for VOC at Cayenne Hospital Center, thoracic or abdominal pain during VOC, a prior history of ACS, and the Hb SS genotype were identified as the strongest independent predictors of progression to ACS. Low baseline hemoglobin also showed a trend toward increased risk but did not reach statistical significance. A patient-level sensitivity analysis was performed, but its interpretation was limited by missing outcome data and sparse or completely separated predictor categories. Moreover, the receiver operating characteristic (ROC) curve demonstrated an area under the curve (AUC) of 0.86, further confirming our predictive model’s strong performance.

The observed incidence of ACS (in 29% of VOC episodes) was higher than previously reported rates in pediatric populations, which generally range from 15% to 25% in single-center studies [12,13].

Although no significant genotypic differences in acute chest syndrome characteristics were observed in the present study, previous studies have suggested that sickle cell genotype may influence the clinical presentation and severity of ACS [6,7,12,13,14].

Hydroxyurea therapy was identified as an independent factor associated with the occurrence of acute chest syndrome in our multivariate analysis. This finding should be interpreted with caution, as it is likely influenced by confounding by indication. In clinical practice, hydroxyurea is preferentially prescribed to patients with more severe sickle cell disease phenotypes, including those with frequent vaso-occlusive crises or previous severe complications. Consequently, patients receiving hydroxyurea may inherently have a higher baseline risk of developing acute chest syndrome than those not receiving this treatment. The observed association therefore probably reflects the underlying severity of the disease rather than a deleterious effect of hydroxyurea itself.

### 4.1. Comparison with Previous Studies

Several studies have reported similar ACS risk factors [12,13,14,15,16,17]. Hb SS genotype has consistently been associated with increased susceptibility due to higher hemoglobin S concentration and propensity for red blood cell sickling. A history of prior ACS has also been identified as a strong predictor, likely reflecting persistent pulmonary vulnerability or recurrent vaso-occlusion. There is a biologically plausible association between thoracic or abdominal pain during VOC and ACS, as these pain sites may indicate rib infarction or early pulmonary involvement predisposing to ACS.

Our study confirms these associations in the context of French Guiana, a region with a high prevalence of sickle cell disease and a substantial burden of disease-related hospitalizations. These epidemiological characteristics provide a relevant setting for evaluating factors associated with acute chest syndrome following vaso-occlusive crises. Acute chest syndrome is a frequent and potentially life-threatening complication of SCD [12]. In our study, its incidence was particularly high. Identifying predictive factors is therefore essential to enable early intervention and prevent potentially fatal outcomes. Ideally, interventions should even be implemented before ACS onset in order to prevent its occurrence, given the severity and high mortality associated with this complication. To date, no validated predictive score is available to reliably anticipate the development of ACS. Although several studies have identified predictive factors [12,13,14,15,16,17] and others have proposed predictive scoring systems [18], all of these studies were observational and no randomized controlled trials have been conducted thus far. Moreover, due to the severity of this complication, randomizing patients to a non-intervention arm in the context of ACS is ethically and practically challenging.

The originality of our study lies in its focus on VOC presenting to the ED—the most common reason for pediatric SCD-related consultations—and in identifying factors associated with progression to ACS. The factors identified in our study, some of which have already been reported in previous studies, further expand and reinforce the spectrum of risk factors that should be considered whenever a child with sickle cell disease presents to the pediatric emergency department with a VOC.

In the presence of at least one of these risk factors, the optimal preventive management strategy for avoiding ACS development remains unclear: should patients receive standard oxygen therapy, high-flow nasal cannula oxygen, or noninvasive ventilation (NIV)? These strategies warrant evaluation in future randomized controlled trials.

### 4.2. Clinical Implications

Identifying predictive factors for ACS has several practical implications.

1. Early risk stratification: Children presenting with VOC characterized by thoracic or abdominal pain, prior ACS, or HbSS genotype should be closely monitored for early signs of pulmonary involvement.
2. Preventive measures: These patients may benefit from enhanced supportive care, including oxygen monitoring, incentive spirometry, and careful fluid management.
3. Therapeutic planning: Recognition of high-risk VOC episodes may guide early transfusion strategies or prompt escalation to intensive care if ACS develops rapidly.
4. Hydroxyurea treatment was more frequent among VOC episodes complicated by ACS than among uncomplicated VOC episodes. In the multivariable analysis, hydroxyurea exposure remained positively associated with ACS occurrence. This finding should not be interpreted as evidence of a harmful effect of hydroxyurea, because treatment was more likely to be prescribed to patients with a more severe underlying sickle cell disease phenotype. Residual confounding by indication is therefore highly probable.

### 4.3. Strengths and Limitations

The strengths of this study include its real-world, hospital-based cohort, comprehensive data collection, and inclusion of all genotypes with confirmed SCD. These findings identify clinical characteristics associated with ACS occurrence and should be considered exploratory pending validation in an independent cohort.

The study’s limitations include its retrospective design, the potential underreporting of clinical events, and the single-center setting, which may limit generalizability. Additionally, some laboratory and inflammatory markers were not consistently available for all episodes. Model performance was assessed on the same dataset used for model development. The reported discrimination and classification measures are therefore apparent estimates and may be optimistic. The model was evaluated using the same dataset on which it was developed, and no bootstrap resampling or cross-validation was performed. Therefore, the reported discrimination, calibration, and classification measures may be optimistic because of overfitting. The model should be considered exploratory until it has undergone formal internal and external validation. The primary analysis treated VOC episodes as separate observations, although some episodes occurred in the same patient. Consequently, standard errors may have been underestimated and confidence intervals may be overly narrow. The patient-level sensitivity analysis only partially addresses this limitation. Despite these limitations, our findings are clinically relevant and support the early identification of children at risk of ACS.

### 4.4. Future Perspectives

Prospective studies are warranted to confirm these predictive factors and to evaluate whether targeted interventions during high-risk VOC episodes can reduce ACS incidence. The integration of biomarkers such as inflammatory or hemolytic parameters could further improve risk stratification. Finally, extending this research to multicenter cohorts in French Guiana and the Caribbean may help to refine preventive strategies and inform public health policies.

## 5. Conclusions

In pediatric patients with SCD hospitalized for VOC, thoracic or abdominal pain, prior ACS, and Hb SS genotype are strong independent predictors of progression to ACS. The early identification of high-risk patients may facilitate timely interventions, improve clinical outcomes, and reduce the length of hospital stay. These findings underscore the importance of tailored monitoring and preventive strategies in regions with high SCD prevalence, such as French Guiana.

## Figures and Tables

**Table 1:** Baseline characteristics of VOC episodes.

Characteristics	Uncomplicated VOC (No1 = 586)	VOC → ACS (No2 = 239)	*p*
Age (median, IQR)	11 [7–15]	15 [11–17]	<0.001
Male sex (%)	261 (45)	138 (58)	0.001
Hb SS/S/ß° genotype (%)	398 (68)	182 (76)	<0.001
Hydroxyurea therapy (%)	232 (40)	197 (82)	<0.001
History of acute chest syndrome (%)	112 (19)	122 (51)	<0.001
Comorbidity (%)	206 (35)	104 (44)	0.02
Cholecystectomy history (%)	152 (26)	70 (29)	0.3
Splenectomy history (%)	68 (12)	15 (6)	0.02
Baseline hemoglobin < 8 g/dL (%)	85 (16)	21 (10)	0.05
Thoracic/abdominal pain VOC (%)	354 (60)	231 (97)	<0.001
Length of Hospital Stay > 5 days (%)	103 (17)	34 (15)	0.3
Current white blood cells (median, IQR)	16 [11–21]	16 [11–20]	0.6
Current neutrophils (median, IQR)	10 [7–15]	10 [7–14]	0.9
Current platelets (median, IQR)	400 [400–400]	400 [400–400]	
Current reticulocytes (median, IQR)	244 [166–350]	180 [118–270]	0.1
Current LDH (median, IQR)	650 [538–800]	650 [490–800]	0.3
Current CRP (median, IQR)	28.5 [4–128]	71.5 [7–180]	0.09

“No1” here is the episode of VOC counts in the Uncomplicated VOC column, and “No2” in the VOC -> ACS column is the count of ACS episodes.

**Table 2:** Univariate analysis.

Variable	OR (95 CI%)	*p*-Value
Age (per year)	2.8 (2.1–3.7)	<0.001
Male sex	1.7 (1.3–2.3)	0.001
Hb SS/S/ß° genotype	3.4 (2.3–5.1)	<0.001
Thoracic or abdominal pain VOC	18.9 (9.2–39.1)	<0.001
History of ACS	4.5 (3.2–6.2)	<0.001
Hydroxyurea therapy	7.2 (4.9–10.3)	<0.001
Baseline Hb < 8 g/dL	0.6 (0.4–1.0)	0.05
Comorbidity history	1.4 (1.04–1.9)	<0.03
Length of Hospital Stay	1.7 (1.2–2.6)	0.004

VOC: vaso-occlusive crisis.

**Table 3:** Multivariable Analysis.

Characteristics	Uncomplicated VOC (No1 = 586)	VOC → ACS (No2 = 239)	aOR (95%CI)	*p*
Genotype Hb SS/S/ß° (%)	398 (68)	182 (76)	1.8 (1.2–2.4)	<0.001
Hydroxyurea therapy (%)	232 (40)	197 (82)	8.7 (5.2–14.5)	<0.001
History of acute chest syndrome (%)	112 (19)	122 (51)	7.4 (4.5–12.1)	<0.001
Thoracic/abdominal pain VOC (%)	354 (60)	231 (97)	14 (6–32)	<0.001
Age (years)			1.6 (1.1–2.4)	0.02
0–5	101 (17)	8 (3)		
5–10	176 (30) 309 (53)	39 (16) 192 (80)		
>10 Male sex	261 (45)	138 (58)	1.6 (1.1–2.4)	0.04

VOC: vaso-occlusive crisis; ACS: acute chest syndrome; aOR: adjusted odds ratio. “No1” here is the episode of VOC counts in the Uncomplicated VOC column, and “No2” in the VOC -> ACS column is the count of ACS episodes.

## Data Availability

The data that support the findings of this study are available from the corresponding author upon reasonable request, subject to institutional and regulatory approvals and in accordance with data protection regulations.
